# Supplementary material for: Estimation of the Cadmium Nephrotoxicity Threshold from Loss of Glomerular Filtration Rate and Albuminuria
Source: Toxics. 2023 Sep 6;11(9):755. doi: 10.3390/toxics11090755 (PMC10534899; doi:10.3390/toxics11090755)
Supplement: Supplementary file 1 [file toxics-11-00755-s001.zip › toxics-2589361-supplementary.pdf]

Supplemental Material: Estimation of the Cadmium Nephrotoxicity Threshold from Loss of GFR and Albuminuria

Soisungwan Satarug, David A. Vesey, Tanaporn Khamphaya, Phisit Pouyfung, Glenda C. Gobe, and Supabhorn Yimthiang

Table S1: Predictors of eGFR based on cadmium excretion rate normalized to creatinine excretion

Independent variables factors	eGFR, mL/min/1.73 m^2					
	All subjects, *n* = 444		Women, *n* = 331		Men, *n* = 112	
	β	*p*	β	*p*	β	*p*
Age, years	−0.442	<0.001	−0.449	<0.001	−0.408	<0.001
BMI, kg/m^2	−0.043	0.317	−0.021	0.669	−0.132	0.156
Log[(E_{Cd}/E_{cr}) ×10^3, µg/g creatinine	0.042	0.435	0.044	0.476	0.052	0.634
SBP, mmHg	−0.105	0.073	−0.084	0.213	−0.162	0.186
DBP, mmHg	0.007	0.893	−0.001	0.985	0.038	0.747
Gender	−0.038	0.424	−	−	−	−
Smoking	−0.003	0.944	0.000095	0.998	−0.002	0.980
Diabetes	−0.057	0.241	−0.071	0.207	0.007	0.945
Adjusted R^2	0.282	<0.001	0.284	<0.001	0.212	<0.001

eGFR, estimated glomerular filtration rate; β, standardized regression coefficient; adjusted R^2, coefficient of determination. Coding: male, 1, female, 2; non-smoker, 1, smoker, 2; non-diabetic, 1, diabetic, 2. β indicates strength of association of eGFR with eight independent variables (first column). Adjusted R^2 indicates the proportion of eGFR variance explained by all independent variables. For all tests, *p*-values ≤ 0.05 indicate a statistically significant effect of individual independent variables on eGFR.

Table S2. Predictors of albumin excretion rate normalized to creatinine excretion

Independent variables/ factors	Log [(E_{alb}/E_{cr}) ×10^4, mg/g creatinine					
	All, *n* = 473		Women, *n* = 357		Men, *n* = 116	
	β	*p*	β	*p*	β	*p*
Age, years	0.012	0.840	0.047	0.502	−0.027	0.811
BMI, kg/m^2	0.036	0.440	0.051	0.340	0.004	0.964
eGFR, mL/min/1.73 m^2	−0.092	0.078	−0.103	0.090	−0.059	0.575
Log([Cd]$_b$ ×10^3), µg/L	0.136	0.017	0.157	0.016	0.143	0.231
Gender	0.043	0.399	−	−	−	−
Smoking	0.017	0.749	0.062	0.261	−0.082	0.402
Diabetes	0.245	<0.001	0.214	<0.001	0.355	0.002
Hypertension	0.163	<0.001	0.158	0.003	0.171	0.069
Adjusted R^2	0.092	<0.001	0.084	<0.001	0.103	0.008

β, standardized regression coefficient; eGFR, estimated glomerular filtration rate; adjusted R^2, coefficient of determination. Coding: male, 1, female, 2; non-smoker, 1, smoker, 2; non-diabetic, 1, diabetic, 2. β indicates strength of association of $\log[(E_{alb}/C_{cr}\times10^4)$ with eight independent variables (first column). Adjusted R^2 indicates the proportion of $\log[(E_{alb}/E_{cr}\times10^4)$ variance explained by all independent variables. For all tests, *p*-values ≤ 0.05 indicate a statistically significant effect of individual independent variables on $\log[(E_{alb}/E_{cr}\times10^4)$.

Table S3. Prevalence odds ratios for low eGFR and albuminuria in relation to cadmium excretion rate normalized to creatine excretion

Independent variables	Low eGFR		Albuminuria	
	POR (95% CI)	*p*	POR (95% CI)	*p*
Age, years	1.129 (1.071, 1.189)	<0.001	1.027 (0.991, 1.064)	0.147
BMI, kg/m^2	1.018 (0.923, 1.122)	0.725	1.040 (0.972, 1.114)	0.256
Gender	2.106 (0.587, 7.555)	0.253	0.539 (0.263, 1.104)	0.091
Smoking	1.532 (0.485, 4.842)	0.468	0.918 (0.435, 1.939)	0.823
Diabetes	3.054 (1.147, 8.132)	0.025	4.861 (2.303, 1.939)	<0.001
Hypertension	1.239 (0.545, 2.818)	0.609	2.080 (1.170, 3.697)	0.013
Tertile of E_{Cd}/E_{cr}				
Low	Referent		Referent	
Middle	4.596 (1.271, 16.61)	0.020	1.875 (0.901, 3.905)	0.093
High	3.964 (1.176, 13,36)	0.026	1.807 (0.810, 4.033)	0.149

POR, prevalence odds ratio; CI, confidence interval; BMI, body mass index; eGFR, estimated glomerular filtration rate. Albuminuria was defined as albumin to creatinine ratios (ACR) ≥ 20 mg/g for men and ≥ 30 mg/g for women Coding, male 1, female 2; non-smoker 1, smoker 2; non-diabetic 1, diabetic 2; code 1 is referent. Data were generated from logistic regression relating POR for low eGFR and albuminuria to seven independent variables (first column). The cut-off values of E_{Cd}/E_{cr} for the low, middle and high tertiles of Cd burden in men were ≤ 1.41, 1.42–3.57, ≥ 3.58 µg/ g creatinine, respectively. Corresponding cut-off values of E_{Cd}/E_{cr} in women were ≤ 1.97, 1.98-4.59, ≥ 4.60 µg/ g creatinine. For men, arithmetic means (SD) of E_{Cd}/E_{cr} in low, middle and high tertiles were 0.40 (0.50), 2.37 (0.66) and 7.07 (4.11) µg/g creatinine, respectively. For women, arithmetic means (SD) of E_{Cd}/E_{cr} in low, middle and high tertiles were 0.49 (0.62), 3.24 (0.78) and 9.06 (4.96) µg/ g creatinine, respectively. Respective numbers of men (women) in low, middle and high ECd/Ecr tertiles were 37 (110), 39 (111) and 38 (113). For all tests, *p*-values ≤ 0.05 indicate a statistically significant effect of individual independent variables to the POR for low eGFR and albuminuria.
